# Mutational Landscape of Cholangiocarcinoma According to Different Etiologies: A Review

**DOI:** 10.3390/cells12091216

**Published:** 2023-04-22

**Authors:** Simona Tavolari, Giovanni Brandi

**Affiliations:** 1 Medical Oncology Unit, IRCCS Azienda Ospedaliero-Universitaria of Bologna, 40138 Bologna, Italy; giovanni.brandi@unibo.it; 2 Department of Medical and Surgical Sciences, University of Bologna, 40138 Bologna, Italy

**Keywords:** cholangiocarcinoma, risk factors, genomic profiling

## Abstract

Recent next-generation sequencing (NGS) studies on large cohorts of cholangiocarcinoma (CCA) patients have clearly revealed the extreme intra- and inter-tumoral molecular heterogeneity that characterizes this malignancy. The lack of a stereotyped molecular signature in CCA makes the identification of actionable therapeutic targets challenging, making it mandatory to have a better understanding of the origin of such heterogeneity in order to improve the clinical outcome of these patients. Compelling evidence has shown that the CCA genomic landscape significantly differs according to anatomical subtypes and the underlying etiology, highlighting the importance of conducting molecular studies in different populations of CCA patients. Currently, some risk factors have been recognized in CCA development, while others are emerging from recent epidemiological studies. Nevertheless, the role of each etiologic factor in driving CCA genetic heterogeneity still remains unclear, and available studies are limited. In an attempt to shed more light on this issue, here we review the current literature data on the mutational spectrum of this disease according to different etiologies.

## 1. Introduction

In recent years, the employment of next-generation sequencing (NGS) has paved the way for the development of precision medicine in cancer patients, opening the possibility of identifying the full repertoire of tumor-borne genetic alterations from disease onset to progression. In pediatric and hematological tumors, characterized by a low rate of somatic mutations [1,2], NGS technology has been rapidly translated to clinical practice, redefining the diagnostic work-up and treatment paradigm in this patient setting.

In adult solid tumors, the identification of genomic landscape and actionable molecular targets is more challenging, as tumor genetic makeup is constantly reshaped due to the many mutational events and clonal evolution occurring in cancer cells over the disease course; furthermore, in solid cancers, tumor genetic heterogeneity may be significantly influenced by the existence of multiple cells of origin [3].

This scenario is even more complex in the context of cholangiocarcinoma (CCA), which encompasses an extremely heterogeneous group of malignancies arising from the intrahepatic (iCCA) and extrahepatic (eCCA) biliary tree. The molecular heterogeneity of CCA has been observed not only when comparing iCCA and eCCA subtypes, but also among patients with the same cancer type (inter-patient heterogeneity), and even across the different topographic regions of the tumor from the same patient (intra-patient heterogeneity) [4]. Besides the host genetic determinants, CCA genetic heterogeneity is thought to stem from the high number of risk factors associated with this disease, which can target distinct stem cell niches and cells of origin along the biliary tree, thus evoking distinct molecular mechanisms to initiate cell malignant transformation [4]. Epidemiological evidence suggests that some risk factors are common to iCCA and eCCA (primary sclerosing cholangitis, liver fluke infections, congenital biliary diseases, inflammatory bowel disease), whereas others are more associated with iCCA (cirrhosis, hepatolithiasis, HVB and HVC infection, NAFLD/NASH, alcohol, diabetes) or eCCA (cholelithiasis, choledocholithiasis) development [5]. As to iCCA, a link between etiology and histotype has been recently demonstrated; according to the most recent WHO classification, non-biliary cirrhosis and chronic hepatitis associate with the small duct subtype, whereas biliary chronic diseases, cholangitis, hepatolithiasis and liver fluke infections associate with the large duct subtype [6]. Nevertheless, currently the comprehension of the molecular mechanisms underlying CCA carcinogenesis still remains unclear, and fewer studies have investigated the contribution of each etiologic factor in driving CCA genetic heterogeneity. In an attempt to shed more light on this issue, here we review the current available literature data on the mutational spectrum of this disease according to different etiologies.

## 2. CCA Genomic Landscape in Eastern and Western Patients

The epidemiological trend of CCA shows a wide geographical variation worldwide, with a higher incidence in Eastern compared to Western countries [5]. CCA is more frequent in Southeast Asia (up to 113 per 100,000 person-years), with the highest rate registered in Northeast Thailand, where CCA ranks as the most common primary liver cancer, with an incidence reaching 85 per 100,000 person-years [7,8]. In Western countries, CCA is still considered a relatively rare disease (an incidence of less than 6 per 100,000 person-years); nevertheless, the last two decades have witnessed a progressive increase in iCCA incidence and mortality, both in the USA and Europe, while rates for eCCA have remained relatively stable or have even decreased in some European countries [9,10,11,12,13]. Notably, a recent cross-sectional study estimated that the incidence rate of iCCA (along with liver cancer) will further increase in the coming twenty years in the USA, going from 43,000 cases in 2020 to 100,000 cases by 2040, becoming the third most-common cause of cancer-related death (41,000 deaths) [14].

The significant variation in CCA global incidence is thought to reflect a different distribution of the underlying risk factors and genetic backgrounds [5]. In East Asia, where liver flukes are endemic, parasitic infections with Opisthorchis viverrini (O. viverrini) and Clonorchis sinensis (C. sinensis) represent the major risk factor for CCA [5]; a different scenario occurs in Western countries, where primary sclerosing cholangitis (PSC) is the most well-known risk factor for CCA development [5]. Nevertheless, in the majority of Western patients, CCA occurs sporadically, without any identified etiologic factors [5].

The different geographical distribution of CCA risk factors and genetic determinants likely contribute not only to the differences in global incidence of this disease, but also to the different genomic landscape observed between Eastern and Western CCA patients (Table 1).

A genomic study on 164 Chinese patients and 283 patients from the USA reported a significantly higher tumor mutational burden (TMB) in the Asian cohort compared to the Western cohort [15]. The most-recurrent mutated genes in Asian patients were TP53 (41.5%), TERT (13.4%), BRCA1/2 (8.5%), TGFBR2 (6.1%), RBM10 (6.1%), NF1 (6.1%), SPTA1 (5.5%), RB1 (5.5%), KMT2C (5.5%) and DDR (4.9%), whereas a high occurrence of CDKN2A/B (30%), IDH1/2 (23.3%) and BAP1 (17.3%) mutations was found in Western patients [15]. Another recent study comparing the genomic profile of 253 iCCA Chinese patients to that of 158 iCCA patients from USA reported a higher KRAS and a lower IDH1, ARID1A and TERT mutation rate in the Asian cohort, compared to the Western one [16].

## 3. Liver Fluke Infections

Liver fluke infections with O. viverrini and C. sinensis are the leading cause of CCA development in Southeast Asia [5]. O. viverrini is endemic in Thailand, Laos, Cambodia and Vietnam, with some reported cases also in Malaysia, Singapore and the Philippines; C. sinensis is endemic in Northeast China, Southern Korea, Japan, Taiwan and Northern Vietnam [1]. These parasitic diseases represent a serious global health problem, as about 700 million people are at risk of infection worldwide, with more than 45 million people estimated to be affected in the Mekong region only [17]. In humans, infection occurs by the ingestion of raw or partially cooked fish containing encysted metacercariae, the infective stage of the parasite. Following ingestion, metacercariae are digested by gastric and intestinal juices, and juvenile flukes migrate to the intrahepatic bile ducts through the ampulla of Vater and common bile duct; here, adult flukes reproduce and, in the absence of treatment, their lifespan can be as long as 25–30 years [18]. Both O. viverrini and C. sinensis are classified as Group 1 carcinogens to humans by the International Agency for Research on Cancer (IARC) [19]. The mechanism of carcinogenesis driven by these parasites is multifactorial, and includes mechanical/chronic injury to the biliary epithelial cells, chronic tissue inflammation via reactive oxygen intermediates and nitric oxide release, and an increase in cell proliferation via parasite secretion products [20].

The genomic landscape of liver fluke CCAs has been investigated in different studies. In a first study, Ong et al. sequenced 54 cases of fluke-positive CCAs, reporting a high mutation rate in TP53 (44.4%), KRAS (16.7%), SMAD4 (16.7%) MLL3 (14.8%), ROBO2 (9.3%), RNF43 (9.3%), GNAS (9.3%) and PEG3 (5.6%) genes [21]. Another study comparing 108 cases of fluke-positive CCAs to 101 cases of fluke-negative CCAs from Asia and Europe reported a distinct mutational pattern between the two groups. The most-recurrent mutated genes in fluke-positive CCAs included TP53 (39.8% vs. 9.3%), SMAD4 (19.4% vs. 5.8%), ARID1A (17.6% vs. 10.5%) and MLL3 (13% vs. 3.5%), whereas fluke-negative CCAs showed a higher mutation rate in BAP1 (10.5% vs. 2.8%) and IDH1/2 (9.3% vs. 2.8%) genes [22]. Similar findings were found in another exome sequencing study on 32 iCCA Western patients, reporting a high frequency of BAP1 (25%), ARID1A (19%), IDH1/2 (19%) and PBRM1 (17%) mutations in this patient setting [23]. Furthermore, an additional study evaluating the occurrence of FGFR alterations on 121 fluke-positive and 95 fluke-negative CCAs showed that FGFR fusions were strikingly enriched in the fluke-negative cases (11.6%) compared to fluke- positive tumors (1.65%) [24]. More recently, genomic profiling of 133 fluke-positive CCAs and 356 fluke-negative CCAs identified four different clusters of CCA: clusters one and two included fluke-positive CCAs and were enriched in ERBB2 amplifications and TP53 mutations, whereas clusters three and four included fluke-negative CCAs and were enriched in IDH1/2, BAP1 and FGFR rearrangements [25]. Moreover, fluke-positive CCAs were found to harbor significantly more somatic mutations compared to fluke-negative CCAs (median 4700 vs. 3143 per tumor) [25].

Overall, these studies provide a clear evidence of a distinct mutational landscape between fluke-positive and fluke-negative CCAs, with a higher mutation rate in genes associated with genome stability, epigenetic regulation, Wnt signaling, G protein signaling and TGF-β/SMAD4 signaling in the former group of CCAs, and a higher mutation rate in genes associated with chromatin remodeling and metabolic enzymes in the last group of CCAs.

## 4. Primary Sclerosing Cholangitis

Primary sclerosing cholangitis (PSC) is a rare, autoimmune cholestatic disease causing fibro-inflammatory destruction of the intra- and/or extrahepatic bile ducts, often complicated by inflammatory bowel disease, particularly ulcerative colitis [26]; although the disease course may be variable, in most cases it ultimately leads to biliary cirrhosis and liver dysfunction. In Europe and North America, the incidence rate of PSC ranges from 0.5 to 1.3 per 100,000 person-years, whereas in Asian countries this disease is less common [27]. In Western countries, PSC represents the most important risk factor for CCA, and patients with PSC carry a 400-fold higher risk of developing this malignancy compared to the general population [28]. The occurrence of CCA in these patients is higher in the first 2 years after PSC diagnosis, with 30–50% of CCAs developing within the first year [28].

The etiopathogenesis of PSC still remains uncertain, but it is thought to involve a complex combination of host genetics, environmental and immunological factors [29]. Currently, less is known about the mutational spectrum of PSC-associated CCA. A recent study on 26 patients with PSC, including 24 cases with CCA and 2 cases with biliary dysplasia, showed a high intertumor and intratumor heterogeneity in TP53 mutations and CDKN2A gene loss [30]. TP53 mutations were detected in eleven PSC-associated CCAs and three PSC-associated dysplasia samples; in particular, one PSC-associated CCA sample carried two different TP53 missense mutations and one PSC-associated dysplasia sample showed the combination of a splice site and a TP53 missense mutation. Loss of the CDKN2A gene was detected in 8 PSC-associated CCAs and 3 PSC-associated dysplasia samples [30]. However, because of the small sample size of patients analyzed in this study, no definitive conclusions can be drawn about the genomic landscape in PSC-associated CCA. More compelling evidence has been provided by a larger genomic study performed on 60 PSC-associated iCCAs, 64 PSC-associated perihilar CCAs, 18 PSC-associated distal CCAs, 28 PSC-associated gallbladder cancers and 4 PSC-associated tumors of unknown anatomical origin [31]. The most-recurrent mutated genes included TP53 (35.5%), KRAS (28.0%), CDKN2A (14.5%), SMAD4 (11.4%), PIK3CA (9.1%), CDKN2B (8.6%), ERBB2 (8.1%), KDM5A/6A (7.0%) and ROBO1 (7.0%); furthermore, 8.4% of cases were positive for HER2 amplification. Less-frequently-mutated genes (2–5%) were FBXW7, TGFBR2, GNAS, SMARCA4, BRAF and ARID1A; no case with MSI-high was detected among all the BTCs analyzed [31]. Interestingly, no IDH1/2 mutations and FGFR2 translocations, the typical genomic signature of iCCA [32], were detected in the 60 iCCAs included in the study, except for a single case carrying an IDH1-mutation, likely occurring sporadically in this patient. Overall, these findings suggest that the genomic spectrum of PSC-associated CCAs closely resembles that of liver-fluke-positive CCAs. It has been hypothesized that this molecular similarity may be due, at least in part, to a common cellular origin of PSC-associated CCAs and fluke-positive CCAs, as both PSC and the liver fluke infection activate biliary tree stem cells within the peribiliary glands of large intrahepatic and extrahepatic bile ducts [33,34].

## 5. Hepatitis Virus Infection

Hepatitis viruses are well-known risk factors for hepatocellular carcinoma (HCC), and worldwide approximately 56% and 20% of HCC cases are related to HBV and HCV infections, respectively [35]. Recent studies suggest that both HBV and HCV may also represent risk factors for CCA, particularly iCCA [36]. It seems that differences in HBV- and HCV-related CCA risk exist between Asian and Western populations. Indeed, while HBV infection represents a risk factor for CCA in some Asian countries such as China, where it is highly endemic, HCV-related CCAs mostly occur in Western countries and the United States [36]. The molecular mechanisms of HBV- and HCV-related CCA carcinogenesis still remain poorly understood, but it has been hypothesized that they may be similar to those driving HCC carcinogenesis, including a status of chronic inflammation due to the continuous presence of the virus in target tissues, and insertional mutagenesis in host cells of viral DNA, which can directly promote malignant transformation [37]. In HCC carcinogenesis, HBV-DNA insertion is an early event after viral infection and results in DNA damage and genomic instability in infected cells; furthermore, the integration sites of HBV-DNA are not random, but target specific loci of the tumor genome [38].

Recently, the presence of HBV-DNA has been also detected in iCCA tumor samples. As for HCC, recent studies suggest that HBV integration is not a random event, but preferentially occurs in the promoter region of target genes [38]. A molecular analysis of 41 HBV-associated iCCAs identified most recurrent HBV integration events in TERT (10%), ZMAT4 (5%), MET (5%), ANKFN1 (5%), and PLXNB2 (5%) genes [39]. Another molecular analysis of a largest population study of 108 HBV-associated iCCAs reported the occurrence of HBV insertion in 41.7% of cases, with recurrent integration sites in TERT, FN1, FAT2, BRD9, ABCA12 and NBPF20 genes [40]. Notably, HBV genome integrations in the TERT gene were found in 7% of HBV-associated iCCAs and clustered in 87.5% of cases in the promoter region of this gene [40], which represents one of the most frequent sites of HBV insertion also in HCC [41]. Furthermore, hot-spot mutations in the TERT promoter were detected in 11 (10%) of HBV-associated iCCAs and were mutually exclusive with HBV insertional events [40]. Interestingly, molecular comparison of HBV-associated iCCAs with 167 HBV-associated HCCs, 154 non-viral/fluke-negative iCCAs and 16 non-viral/fluke-positive iCCAs revealed that the overall mutational profile of HBV-associated iCCAs was closer (except for the occurrence of the RB1 mutation also detected in HBV-associated HCCs) to that of non-viral/fluke-negative iCCAs, showing recurrent mutation rates in the IDH1, PBRM1 and PIK3CA gene; however, differently to non-viral/fluke-negative iCCAs, which show recurrent FGFR2 genetic aberrations, FGFR2 fusions were detected only in 0.9% of HBV-iCCAs [40]. Furthermore, when HBV-associated iCCAs were stratified according to the presence or absence of HBV-DNA insertion in the tumor genome, a higher frequency of *TP53* mutations was found in the subgroup of iCCAs positive for viral integration [40].

A positive association between the occurrence of TP53 mutations and HBsAg positivity has been also reported in a previous study on 102 iCCA Chinese patients; in the same study, a high frequency of KRAS mutations was detected in patients negative for HBsAg [42]. Similarly, a molecular study on 97 liver cancers with biliary phenotype reported a higher recurrence rate of KRAS (20%) and IDH1/2 (20%) mutations in hepatitis-negative patients, compared to hepatitis-positive patients [43].

Overall, these molecular studies suggest that insertions of the hepatisis virus genome occur frequently in iCCAs patients and that a different mutational pattern exists between iCCAs patients positive for HBV/HCV and iCCAs patients negative for such an infection.

## 6. Bile Duct Cysts

Bile duct cysts are a rare congenital disorder characterized by cystic dilatation of the intrahepatic and/or extrahepatic biliary tree, usually diagnosed during childhood or in young adults. According to Todani’s classification, they can be anatomically classified into type I–V, with type I and IV accounting for about 50–80% and 15–35% of all cases, respectively [44]. The incidence of bile duct cysts is high in Asian countries, especially China and Japan (up to 100 per 100,000 person-years), while is relatively low in Western populations (1:100,000 per 100,000 person-years) [45]. The increase in CCA risk in patients with bile duct cysts is well established (up to 30-fold, compared to the general population), especially in those carrying type I and IV cysts, remaining significant even after cyst excision [46,47]. The molecular mechanisms leading to CCA development in patients with bile duct cysts still need to be fully clarified. It has been hypothesized that reflux of pancreatic enzymes, bile stasis and increased intraductal concentration of bile acids may play a central role in biliary tract carcinogenesis [48]. Indeed, the continuous stimulus of biliary epithelial cells by activated pancreatic enzymes, increased secondary bile acids and other mutagens results in chronic inflammation and increased cell proliferation, which in turn lead to oncogene and/or tumor suppressor gene mutations and cell malignant transformation [48].

Currently, the mutational pattern of CCA associated with bile duct cysts has not been investigated in large population studies. Recently, the case of a 16-year-old girl who developed an eCCA after two years from choledochal cyst resection has been reported [49]. Tumor sequencing showed the occurrence of de novo somatic mutations in TP53 and RBM10 genes, along with KRAS amplification; however, these genetic alterations were likely sporadic in this patient, as they occurred shortly after cyst resection [49].

Bile duct cysts are frequently associated with pancreaticobiliary maljunction, a congenital malformation where pancreatic and bile ducts join anatomically outside the duodenal wall [50]. Pancreaticobiliary maljunction is widely recognized as an important risk factor for biliary tract cancer [51]. Biliary tract carcinogenesis associated with pancreaticobiliary maljunction is characterized by a hyperplasia–dysplasia–carcinoma sequence induced by the status of chronic inflammation in the biliary epithelium [51]. A molecular study on cancerous and noncancerous biliary tract epithelium from 37 patients with pancreaticobiliary maljunction reported a high incidence rate of KRAS and TP53 mutations both in cancerous and noncancerous lesions, suggesting that in this patient setting the occurrence of these mutations may represent an early event in biliary carcinogenesis [52]. Despite these findings, no definitive conclusion can be drawn about the genetic profile of CCA associated with bile duct cysts, and further molecular studies on a larger patient population are needed.

## 7. Liver Cirrhosis

Cirrhosis represents the final stage of liver fibrosis and is characterized by profound changes in the hepatic architecture, with the formation of fibrous septae and regenerative nodules in response to chronic liver injury, which progressively leads to liver disfunction. Cirrhosis represent the main risk factor for HCC, but epidemiological studies have proven evidence that it also represents a risk factor for CCA, especially iCCA [53]. A recent large-scale retrospective study on 1312 iCCA Asian patients reported that 23.0% of iCCA cases occurred on a cirrhotic liver, a much higher percentage than in Western countries [54]; furthermore, 90.1% of cirrhotic patients showed HBsAg positivity, indicating that liver cirrhosis is mostly associated with HBV infection in China [54].

The molecular mechanisms underlying iCCA carcinogenesis in cirrhotic patients are not well understood, but are thought to be similar to those driving HCC development. Regenerative nodules encountered in liver cirrhosis are sustained by ductular reaction, which is characterized by the appearance of reactive ductules comprising hepatic progenitor cells and transit-amplifying cells committed towards hepatocyte and/or cholangiocyte lineages [55].

Differently from HCC, most iCCAs occur in non-cirrhotic liver [56], and studies that have specifically examined the molecular profile of iCCAs occurring in cirrhosis are very limited.

Genomic profiling of 10 iCCAs developed on cirrhotic liver reported the occurrence of CDKN2A gene mutation or deletion in 40% of cases, IDH1/2 mutation in 30% of cases, and FGFR2 translocation/mutation in 20% of cases; in addition, 40% of cases carried mutations in chromatin regulator genes PBRM1 (20%), ARID1A (10%) and BAP1 (10%) [57]. TERT promoter mutation was detected only in one case, and another case showed a TP53 mutation and concomitant loss of chromosome 17p. Overall, 50% of cases showed the loss of chromosome 3p, which was associated with BAP1 and/or PBRM1 mutation in three cases [57].

## 8. Hepatolithiasis

Hepatolithiasis refers to the presence of stones within the intrahepatic bile ducts. The incidence of hepatolithiasis is low in Western countries (from 0.6% to 1.3%), but relatively high in China, Taiwan, Hong Kong, Korea and Japan (from 2% to 25%), where it is frequently related to liver fluke infection with C. sinensis [58]. The association between hepatolithiasis and CCA development is well-documented [5], with an overall CCA incidence of 5–13% in patients with this pathological condition [59]. The link between hepatolithiasis and CCA development is not fully understood, but chronic inflammation (mainly related to recurrent cholangitis, bile stasis, biliary stricture and bacterial infection, which often occur in patients with hepatolithiasis), likely plays a central role in biliary carcinogenesis [58].

CCA carcinogenesis associated with hepatolithiasis is thought to follow a stepwise progression from a precancerous lesion, namely biliary intraepithelial neoplasia (BilIN), to invasive CCA [60]. BilIN is classified as BilIN-1 (corresponding to low-grade dysplasia), BilIN-2 (corresponding to high-grade dysplasia) and BilIN-3 (corresponding to carcinoma in situ) [60]. A study on patients with hepatolithiasis, including 3 cases without BilIN lesions, 12 cases with BilIN-1, 16 cases with BilIN-2, 10 cases with BilIN-3 and 38 cases with iCCAs, detected KRAS mutations in 48% of patients with BilIN (but not in those without BilIN lesions) and in 31.5% of iCCAs [61]. Furthermore, the prevalence of KRAS mutations was highest in BilIN-2 lesions (43.8%), compared to BIlIN-1 (25%) and BilIN-3 (30%), suggesting that this genetic alteration likely occurs early during the progression from BilIN to iCCA [61].

## 9. Thorotrast

Thorotrast is a radioactive colloidal suspension of thorium dioxide that has been used from the 1930s to the 1950s as a radiographic contrast agent. Once intravascularly injected, it remains in the reticuloendothelial system for many decades, thus accumulating in different organs, mainly the liver [62]. Thorotrast is a well-known risk factor for primary liver cancers, particularly iCCA [5]. Subjects exposed to this agent have indeed a 300-fold increase in iCCA risk [63]. The molecular mechanisms of Thorotrast-induced carcinogenesis have not been fully elucidated; however, as it has a very long half-life in target organs (up to 400 years) and emits alpha-radiations, it is biologically conceivable that the mechanisms may be linked to mutagenic events in oncogenes and tumor suppressor genes. A study on 22 Thorotrast-associated iCCAs reported a high occurrence of TP53 mutations (27.2% of cases), most commonly A-G transitions, in these patients; of note, *TP53* mutations tended to accumulate in advanced tumors [64]. Interestingly, TP53 mutations were also detected in the surrounding normal liver parenchyma where Thorotrast accumulated during the years [64]. Overall, these findings show that Thorotrast continuously damages the DNA of hepatocytes, resulting in A-G transitions of the *TP53* gene; however, as this compound has been banned since 1969, currently the number of iCCAs linked to exposure to Thorotrast is negligible.

## 10. Aflatoxins

Aflatoxins are mycotoxins produced by Aspergillus fungi, which contaminate food. The risk of exposure to afloxins is high in areas where food preservation is sub-optimal, as occurs in several West African countries [65]. The most toxic aflatoxin detected in contaminated food is aflatoxin-B1 (AFB1), classified as a Class 1 carcinogen by the IARC [66]. The liver is the major site of AFB1 detoxification, where it is metabolized into highly reactive epoxides able to form AFB1-DNA adducts, mainly G:C → T:A transversions [67]. It has been shown that the third base of codon 249 (AGG to AGT) in the TP53 gene is a preferential site for AFB1-DNA adduct formation, which results in the aminoacidic substitution of Arginine for Serine (R249S) [67,68]. Chronic exposure to AFB1 is strongly associated with HCC development, and the detection of the TP53 R249S mutation is considered a molecular hallmark of HCC carcinogenesis induced by aflatoxins [68]. In high aflatoxin-exposed areas of Southeast Asia, China and sub-Saharan Africa, this mutation occurs in up to 75% of HCCs, whereas in regions where aflatoxin exposure is low, such as Europe and the USA, TP53 R249S mutation drops down to <6% of HCCs [68].

Conversely, when compared to HCC, the role of aflatoxin exposure in iCCA development still remains unsettled. Sequencing analysis on iCCA Asian patients negative for liver fluke infection reported the occurrence of TP53 mutations in 39 (38.2%) out of 102 cases, a frequency much higher than that reported in other cohorts of iCCA fluke-negative patients from other countries (ranging from 6% to 9.8% of cases) [23,42]. Interestingly, although most of the TP53 mutations identified were truncating, 10 occurred at the codon 249 (R249S) [42]. This represents the first study reporting the occurrence of TP53 R249S mutations in iCCA patients, suggesting that aflatoxin exposure could represent a risk factor not only for HCC, but also for iCCA development in Chinese patients. Despite this hypothesis being consistent with the widespread aflatoxin contamination in Southern China, it requires further studies to be confirmed.

## 11. Organic Solvents

To date, the role of environmental risk factors in CCA development has been little investigated. An increased iCCA incidence has been reported among workers of a printing company in Osaka, following chronic exposure to high concentrations of volatile organic solvents, mainly 1,2-dichloropropane (1,2-DCP) and dichloromethane (DCM) [69]. Among the 111 workers, 17 developed an iCCA at a younger age than the general population (from 25 to 45 years old), and none of them resulted in being exposed to other known risk factors for this disease [69]. Notably, it was observed that iCCA incidence increased with cumulative exposure to 1,2-DCP (adjusted RR = 14.9, 95% CI 4.1–54.3 for middle-exposure category, and adjusted RR = 17.1, 95% CI 3.8–76.2 for high-exposure category), suggesting an exposure–response relationship [70]. The potential association between CCA development and 1,2-DCP and/or DCM exposure has been reinforced by further epidemiological studies reporting the occurrence of this malignancy in an additional 13 printing workers of other Japanese companies [71,72] and in 6 out of 11 Thai workers occupationally exposed to these substances [73].

According to the latest IARC classification, 1,2-DCP is classified as a Group 1 (carcinogenic to humans) carcinogen, while DCM as a Group 2A (probably carcinogenic to humans) carcinogen [74]. The underlying molecular mechanisms linking the exposure to these solvents to CCA carcinogenesis still remain to be fully clarified. Whole-exome sequencing on iCCA tissue samples from four printing workers with long-time exposure to 1,2-DCP revealed a high mutational burden, with a frequency of somatic mutations 30-fold higher than that observed in common iCCA tissue samples, used as controls [75]. Increased C:G to T:A transitions with substantial strand bias, and unique trinucleotide mutational changes (GpCpY to GpTpY and NpCpY to NpTpY or NpApY) were found in all four cases, suggesting exposure to a common strong mutagen [75]. Furthermore, a high recurrence rate of CDKN2A, AXIN1A and ARID1A gene mutations were detected in iCCA-exposed workers, whereas KRAS and SMAD4 were more frequent in common iCCAs [75]. Despite the very small sample size of the patients analyzed, this study suggests that inhaled 1,2-DCP may reach the biliary epithelium and induce DNA damage in targeted cells, leading to malignant transformation; however, further studies are required, to confirm these preliminary findings.

## 12. Asbestos

Asbestos is a natural mineral that has been widely used in industry during the past century and is classified by IARC as carcinogenic to humans (category 1) [76]. Despite being banned in 52 countries, the health risks continue to be relevant, since about 125 million people worldwide are still environmentally exposed to this carcinogen and the growth rate of asbestos-related cancers is expected to increase in the coming years [77], due to the long latency period between exposure and disease development.

Asbestos-induced carcinogenesis is complex, and involves different mechanisms, including chronic inflammation, reactive oxygen/nitrogen species production, induction of chromosomic/genomic aberrations, immune response reduction, absorption of carcinogens and ionizing radiations, and binding to nucleic acids and nuclear proteins [78]. The susceptibility to asbestos-induced carcinogenesis seems to vary among the different tissue types, making some organs at a higher cancer risk compared to others [79]; as for the liver, the accumulation of asbestos fibers can be facilitated by the high microvascular permeability of the hepatic sinusoids [80].

In recent years, the possible role of asbestos exposure and increased iCCA risk has been reported in some case-control studies. The first study, carried out on a cohort of 155 Italian CCA patients matched to controls, found a fourfold increased risk of iCCA in subjects exposed to asbestos, whereas limited evidence was found for eCCA (OR = 2.09, 95% CI 0.83–5.27) [81]. Similarly, a second study nested in the Nordic Occupational Cancer (NOCCA) cohort and including 5430 CCA cases, reported an increased risk of CCA in subjects exposed to asbestos, with a stronger association for iCCA than eCCA [82]. More recently, a cross-sectional study in Thai CCA patients reported that 11 (5.5%) out of 200 cases had a possible occupational cause, with 6 (54.5%) cases related to asbestos exposure [73].

In an exploratory study, asbestos fibers were detected in five (71%) out of seven Italian iCCA patients from Casale Monferrato, an area with high asbestos exposure [83]. Interestingly, another study showed that asbestos exposure is more frequently associated with the small-bile-duct iCCA histotype, rather than the large-duct histotype, suggesting that asbestos fibers may represent a parenchymal, rather than a ductal risk factor for iCCA development [84].

Genomic profiling of 22 iCCA patients, of which 10 resulted as exposed and 12 not-exposed to asbestos, according to the Italian National Mesothelioma Register (ReNaM) questionnaire for asbestos exposure, revealed a significantly higher frequency (27%) of BAP1 somatic mutations in asbestos-exposed patients, compared to not-exposed (5%) [85]. Moreover, the development of an iCCA was recently reported in a 47 year-old patient without any known CCA risk factors, except for occupational exposure to low levels of asbestos; of note was the fact that the patient carried a BAP1 germline mutation, along with BAP1 loss of heterozygosity in tumor cells, in line with the classical two-hit model of tumor suppressor genes [86].

Overall, these findings reinforce the notion of a putative causal role of asbestos in iCCA carcinogenesis, deserving further investigation in future studies.

## 13. Conclusions

The use of NGS has led to an unprecedented understanding of CCA tumor biology, unravelling the complex and heterogeneous genomic landscape of this disease. Despite this progress, many critical questions still remain open and need to be defined, including a better identification of the risk factors associated with this disease, especially in Western patients, and their role in contributing to CCA genetic heterogeneity. Available molecular profiling of CCAs arising from different etiologies have shown different clusters of genomic alterations, converging into different functional categories mainly involving chromatin remodeling (ARID1A, BAP1, PBRM1 mutations), oncogenic addiction (KRAS, ERBB2, FGFR2 mutations) and metabolic reprogramming (IDH1/2 mutations) (Table 2). In the coming years, improved knowledge of CCA risk factors and associated molecular mechanisms is expected not only to enable the development of more effective therapeutic strategies, but also to facilitate the surveillance procedures for cohorts of subjects exposed to such risk factors and at high risk of disease. Progress in the management of CCA will require a close collaboration between basic science and clinical research in the near future, in order to provide more effective measures that might modify the course of this aggressive and worrying malignancy for the better.

## Figures and Tables

**Table 1:** Comparison of Western and Eastern CCAs.

	Incidence Rate	Most Associated Risk Factor	N° of Cases Analysed for Each Study	Genomic Landscape	Ref.
**Western CCAs** **vs.** **Eastern CCAs**	˂6 per 100,000 person-years vs. up to 113 per 100,000 person-years (Southeast Asia)	Primary sclerosing cholangitis vs. Liver fluke infection	1. 283 patients from USA vs. 164 Chinese patients	1. CDKN2A/B (30%), IDH1/2 (23.3%) and BAP1 (17.3%) mutation vs. TP53 (41.5%), TERT (13.4%), BRCA1/2 (8.5%), TGFBR2 (6.1%), RBM10 (6.1%), NF1 (6.1%), SPTA1 (5.5%), RB1 (5.5%), KMT2C (5.5%) and DDR (4.9%) mutation	[15]
			2. 158 iCCA patients from USA vs. 253 iCCA Chinese patients	2. IDH1 (28%) and ARID1A (20%) mutation vs. KRAS (18%) mutation	[16]

**Table 2:** Mutation signature of CCA according to different etiologies.

Etiology	Geographic Distribution	N° of Cases Analyzed for Each Study	Mutation Signature	Ref.
**Liver fluke infection**	Southeast Asia, mainly Mekong region	1. 54 fluke-positive CCAs	1. TP53 (44.4%), KRAS (16.7%), SMAD4 (16.7%) MLL3 (14.8%), ROBO2 (9.3%), RNF43 (9.3%), GNAS (9.3%) and PEG3 (5.6%) mutation	[21]
		2. 108 fluke-positive CCAs	2. TP53 (39.8%), SMAD4 (19.4%), ARID1A (17.6%) and MLL3 (13%) mutation	[22]
		3. 133 fluke-positive CCAs	3. ERBB2 amplification, TP53 mutation	[25]
**Primary sclerosing cholangitis**	Europe, North America	1. 24 PSC-associated CCAs	1. TP53 (45.8%) mutation, loss of CDKN2A (33.3%)	[30]
		2. 60 PSC-associated iCCAs; 64 PSC-associated perihilar CCAs; 18 PSC-associated distal CCAs; 28 PSC-associated gallbladder cancers; 4 PSC-associated tumors of unknown anatomical origin	2. TP53 (35.5%), KRAS (28.0%), CDKN2A (14.5%), SMAD4 (11.4%), PIK3CA (9.1%), CDKN2B (8.6%), ERBB2 (8.1%), KDM5A/6A (7.0%) and ROBO1 (7.0%) mutation; HER2 amplification (8.4%)	[31]
**Hepatitis B virus**	Asian countries, mainly China	1. 41 HBV-associated iCCAs	1. HBV integration in TERT (10%), ZMAT4 (5%), MET (5%), ANKFN1 (5%), PLXNB2 (5%) genes	[39]
		2. 108 HBV-associated iCCAs	2. HBV integration in TERT, FN1, FAT2, BRD9, ABCA12 and NBPF20 genes; IDH1, PBRM1 and PIK3CA mutation; TP53 mutation in iCCAs positive for viral integration	[40]
		3. 102 HBV-associated iCCAs	3. TP53 mutation	[42]
**Bile duct cysts**	Asian countries, mainly China and Japan	1. 37 BTCs associated with pancreaticobiliary maljunction	1. KRAS and TP53 mutation	[52]
**Liver cirrhosis**	Asian countries, mainly China	1. 10 iCCAs	1. CDKN2A gene mutation/deletion (40%), IDH1/2 mutation (30%), FGFR2 translocation/mutation (20%), PBRM1 (20%), ARID1A and BAP1 (10%) mutation	[57]
**Hepatolithiasis**	China, Taiwan, Hong Kong, Korea and Japan	1. 38 iCCAs	1. KRAS mutation (31.5%)	[61]
**Thorotrast**	Worldwide	1. 22 iCCAs	1. TP53 mutation (27.2%)	[64]
**Aflatoxin B1**	Southeast Asia, China and sub-Saharan Africa	1. 102 iCCAs	1. TP53 R249S and TP553 truncating mutation (38.2%)	[42]
**Organic solvents** (**1,2-dichloropropane**)	Japan and Thailand	1. 4 iCCAs from occupationally-exposed printing workers	1. ARID1A and AXIN1A mutation (75%), CDKN2A mutation (50%)	[75]
**Asbestos**	Western countries	1. 10 iCCAs from occupationally-exposed workers	1. BAP1 mutation (27%)	[85]
		2. 1 iCCA from a patient with low exposure	2. BAP1 germline mutation, BAP1 loss of heterozygosity in tumor cells	[86]
